# Relative Trunk Adipopenia Is Associated with the Severity of Liver Disease and Outcome in Patients with Cirrhosis

**DOI:** 10.3390/jcm15103697

**Published:** 2026-05-11

**Authors:** Aikaterini Kamiliou, Magdalini Adamantou, Eleni Pergantina, Nikolaos Rachiotis, Triada Bali, Dimitrios Mouziouras, Dimitra Lakiotaki, Vasileios Lekakis, Alexandra Alexopoulou, George V. Papatheodoridis, Evangelos Cholongitas

**Affiliations:** 1 First Department of Internal Medicine, Medical School, National and Kapodistrian University of Athens, 11527 Athens, Greece; 2 First Department of Gastroenterology, General Hospital of Athens “Laiko”, Medical School, National and Kapodistrian University of Athens, 11527 Athens, Greecegepapath@med.uoa.gr (G.V.P.); 3 Second Department of Internal Medicine, “Hippokration” General Hospital of Athens, Medical School, National and Kapodistrian University of Athens, 11527 Athens, Greece

**Keywords:** adipopenia, body composition, prognosis, end-stage liver disease, fat, adipose tissue, liver transplantation, sarcopenia, frailty

## Abstract

**Background/Objectives**: Regional adipose tissue has been studied as a prognostic factor in various extra-hepatic diseases, but data in the setting of cirrhosis is scarce. Our aim was to evaluate the association of regional adipose tissue indices with the severity of liver disease and its impact on the outcome of patients with cirrhosis. **Methods**: Three hundred and thirty-seven patients with cirrhosis were prospectively enrolled in the study. Clinical and laboratory data were recorded, and MELD-Na and Child-Turcotte-Pugh scores were calculated. Physical performance was evaluated with handgrip strength, the Short Physical Performance Battery test, and the Liver Frailty Index. Dual-energy X-ray absorptiometry was used for the evaluation of total and regional lean and fat mass. **Results**: A low trunk fat percentage was found to be independently associated with a MELD-Na score ≥ 15 (Odds Ratio: 0.92, *p* = 0.007). MELD-Na score and trunk fat percentage were the only factors independently associated with mortality [Hazard Ratio (HR): 1.078, *p* = 0.02 and HR: 0.95, *p* = 0.03, respectively], while a separate analysis based on gender confirmed this finding only in men. In the total cohort, patients with a trunk fat percentage < 27.5% had worse outcomes (log-rank 11.4; *p* < 0.001). **Conclusions**: This study marks the first effort to examine the association of indices related to regional adipose tissue distribution with the severity of liver disease and outcome. Trunk fat percentage was the only body composition parameter independently associated with advanced liver disease and prognosis in the total cohort specifically in men, but not in women. Further studies are needed to validate the predictive role of adipose tissue in patients with cirrhosis.

## 1. Introduction

Identifying prognostic factors for patients with cirrhosis is of paramount importance in order to predict the risk of death and the need for liver transplantation (LT) [1]. The Child-Turcotte-Pugh (CTP) [2] and Model for End-Stage Liver Disease (MELD) [3] [or, recently, the MELD-Sodium (MELD-Na)] score [4] are commonly utilized to evaluate the prognosis of patients with end-stage liver disease. While these scores have been widely used for years, there is a necessity for the development of new prognostic indicators and factors for cirrhosis [1]. Considering this, alterations in body composition have been studied as an important prognostic factor reflecting nutritional status and risk of liver-related complications [5]. For example, generalized skeletal muscle mass loss [6] has been closely linked to the severity of liver dysfunction and worse outcomes including an increased rate of infection, prolonged hospital stay, hepatic encephalopathy (HE), and reduced quality of life and survival before and after LT [7]. Whereas low muscle mass has been widely studied in patients with cirrhosis, little is known regarding the prognostic impact of other body composition indices, such as total or regional adipose tissue in this group of patients. Adipose tissue comprises 20% to 28% of body mass in healthy individuals and its role depends on its distribution and location [8]. In particular, adipose tissue regulates energy balance and functions as an endocrine organ, secreting hormones that affect energy status and various physiological processes like immunity, reproduction, and body temperature regulation [9,10].

The evaluation of skeletal muscle and adipose tissue is usually based on computed tomography (CT), magnetic resonance imaging (MRI) and dual-energy X-ray absorptiometry (DXA) [11]. CT and MRI have been widely used for the assessment of muscle mass, as well as of subcutaneous and/or visceral fat at the level of the third lumbar vertebrate, and certain studies have shown that both types of adipose tissue are associated with mortality in patients with cirrhosis [12,13]. DXA is a validated tool to investigate body composition phenotypes as it precisely analyses the amount and distribution of fat and lean mass in the whole body, as well as in specific anatomical regions [1], offering insights into their distribution across the limbs and trunk [14]. Literature studies have revealed the impact of regional fat distribution on the outcomes of several extra-hepatic diseases, such as type 2 diabetes mellitus and cardiovascular diseases, using DXA scans [15,16]. However, in the setting of cirrhosis, no study has evaluated the association of DXA-derived regional adipose tissue parameters with the severity of liver disease and their prognostic impact in patients with end-stage liver disease.

Thus, the aim of our study was to analyze, for the first time, the total and regional adipose tissue indices using DXA scanning and to explore their association with the severity of liver dysfunction and their predictive role in patients with cirrhosis.

## 2. Materials and Methods

### 2.1. Study Design and Population

This was a retrospective/prospective study with prospectively collected data. All consecutive adult (≥18 years old) patients with clinically stable cirrhosis attending our liver clinic from March 2018 to September 2024 were included. Patient selection and inclusion/exclusion criteria are presented in Supplementary Section S1. Patient follow-up continued until January 2025, or until death or LT in order to evaluate their outcome from the baseline. The study protocol was approved by our Institutional Review Board (approval number: 307/April 2021) and conformed with the 2017 Declaration of Helsinki. Written informed consent was obtained from all patients.

### 2.2. Clinical Data and Laboratory Measurements

At baseline, a concise medical history was gathered from each patient enrolled in the study, including demographic and clinical characteristics as shown in Supplementary Section S2. The severity of liver disease was evaluated by estimating the CTP [2] and MELD-Na scores [4]. The latter is considered a more objective score compared to the CTP score, and patients with cirrhosis and a MELD-Na score ≥ 15 have a relatively high risk of mortality and need for LT [17].

### 2.3. Evaluation of Physical Function and Frailty

Skinfold calipers were used to measure the triceps skinfold (TSF), along with midarm circumference and midarm muscle circumference (MAMC) (values ≤ 21.5 cm for women and ≤24.2 cm for men were used to define presence of muscle mass depletion) [18]. Hand grip strength was assessed by measuring the static force exerted on a dynamometer, with results reported in kilograms (cut-off < 27 kg for men and <16 kg for women for low grip strength) [19]. The frailty status of each patient was evaluated using the Liver Frailty Index (LFI). Patients with a LFI score > 4.4 were categorized as frail, patients with a LFI score between 3.2 and 4.4 were considered pre-frail, whereas scores < 3.2 defined robust status [20]. The Short Physical Performance Battery (SPPB) score was employed for evaluating the physical performance status using 4 m gait speed, a chair sit-to-stand repeated five times, and a balance test [18]. A SPPB score less than 10 identifies low physical performance [18].

### 2.4. DXA-Based Assessment of Sarcopenia and Adipose Tissue

All participants underwent DXA [Horizon W (Hologic Inc., Marlborough, MA, USA; S/N 300472M)] using standard manufacturing protocols, which provided data on lean and fat mass amount and distribution. DXA was performed in fasting patients and with effort for optimal control of ascites (e.g. including paracentesis in the previous days), while DXA software (APEX version 5.6) was not adjusted for ascites. Regional fat mass evaluation included measurements of the left and right arms and legs, head, as well as trunk (i.e., the visceral and subcutaneous fat located from the pelvic region to the top of the neck but not including the arms). These fat indices were expressed in grams (g) and percentages [the % of fat mass relative to total mass (i.e., fat mass (g)/total mass (g) × 100) for each anatomical region]. Similar measurements in grams (g) were obtained for lean mass in these anatomical regions. Relative trunk adipopenia was considered as a reduced trunk fat percentage measured by DXA. Additionally, in the adipose indices section, variables included visceral and subcutaneous fat mass and fat mass index (FMI) [fat mass per height squared (kg/m^2^)]. Visceral and subcutaneous fat were assessed automatically in the region outlined by the top of the iliac crest and with upper height equivalent to 20% of the distance from the top of the iliac crest to the base of the skull. Finally, in the lean indices section, lean mass index (LMI) [lean mass per height squared (kg/m^2^)] and appendicular lean mass index (ALMI) [appendicular lean mass per height squared (kg/m^2^)] were calculated [14].

### 2.5. Sample Size Calculation

A formal a priori sample size calculation was not performed because the study was based on all consecutive, eligible patients with cirrhosis who attended our liver clinic between March 2018 and September 2024. Thus, the sample size was determined by feasibility rather than by pre-specified assumptions. However, a posteriori power analysis was undertaken to assess whether the available number of events was adequate for the primary analysis.

### 2.6. Statistical Analysis

Categorical variables were expressed by absolute (*n*) and relative (%) frequencies and their ratios were compared by corrected chi-square or two-sided Fisher’s exact tests. Quantitative variables were presented as mean or median with ranges for normally or non-normally distributed data, respectively, and their comparisons were performed using Student’s *t* or Mann–Whitney *U* tests, respectively, while multivariable logistic regression analysis was used to identify the independent variables associated with the severity of liver disease. Multivariate analysis was performed by stepwise logistic regression models including all variables with significant associations (*p* < 0.05) in the univariate analyses. Patient survival was calculated by Kaplan–Meier curves and its associations were assessed using the log-rank test. Univariate and multivariable competing analyses were performed with death as the primary outcome and LT as the competing event using the Fine-Gray model to estimate the effect of covariates on death while accounting for the competing risk of LT [21]. All factors with *p* < 0.05 in the univariate Cox regression analysis were entered into the multivariate model. To assess the discriminative ability to predict the outcome of patients, time-dependent receiver operating characteristic (ROC) analysis was performed, and time-dependent area under the curve (AUC) values were calculated at specified time points [22]. To determine the best cut-off point for the time-dependent ROC analysis, Youden’s Index that optimized the balance between sensitivity and specificity was calculated as the sum of sensitivity and specificity minus one. The threshold corresponding to the maximum Youden’s Index was selected as the best cut-off point. Additionally, Harrell’s C-statistic was computed for each Cox regression model to evaluate the overall discriminative performance across the entire follow-up period [23]. Two-sided *p*-values < 0.05 were considered to be statistically significant. Multicollinearity was assessed using Variance Inflation Factors (VIFs). A VIF < 5 was considered acceptable. Statistical analysis was performed using SPSS (version 29.0, IBM Corp., Armonk, NY, USA) and appropriate R packages within R software (version 4.3.1, R Foundation for Statistical Computing, Vienna, Austria).

## 3. Results

A total of 345 consecutive patients with clinically stable cirrhosis were evaluated, but eight patients were excluded due to the need for combined kidney and liver transplantation and/or previous LT. Thus, 337 patients were included in the study [225 (67%) males, mean age 56 years [standard deviation (SD): 11], 263 with decompensated cirrhosis]. Their baseline characteristics, including those related to physical function/frailty and body composition, are shown in Table 1 and Supplementary Section S3. The therapeutic/large volume paracentesis of ascites was performed one to two days before DXA whenever necessary. Thus, among those with ascites (*n* = 187), 156 had grade 1 ascites at baseline evaluation (i.e., DXA performance).

The comparison between males and females is presented in Table 2. Generally, females, compared to males, had significantly higher limb fat indices and significantly lower lean indices. Interestingly, females had significantly lower serum creatinine compared to males [0.74 (0.2) vs. 1.01 (0.4), *p* < 0.001], possibly due to lower muscle mass. However, all other variables associated with the severity of liver dysfunction were similar between the two genders. As a result, the MELD-Na score was lower in females, compared to males, although this difference was not significant [13.2 (5) vs. 15.7 (6.5), *p* = 0.08] (Table 2).

### 3.1. Characteristics of Compensated vs. Decompensated Patients

Our cohort was divided into two groups: the first group included compensated patients (*n* = 74, 22%) and the second group was composed of decompensated patients (*n* = 263, 78%). The characteristics of the two groups are presented in Table 3. Among DXA-derived body composition indices, the second group had significantly lower percentages of fat in the trunk compared to the former group (31% vs. 33%, *p* = 0.012) (Table 3). In multivariate logistic regression analysis (excluding the components of CTP and MELD-Na scores), MELD-Na score was the only independent factor significantly different between compensated and decompensated patients [Odds Ratio (OR) 1.44, 95% Confidence Interval (CI): 1.24–1.68, *p* < 0.001].

### 3.2. Characteristics of Patients Based on MELD-Na Score

Our cohort was divided into two groups: the first group included patients (*n* = 242, 71.4%) with a MELD-Na score < 15 and the second group comprised patients (*n* = 95, 28.6%) with a MELD-Na score ≥ 15. The characteristics of the two groups are presented in Table 4 and Supplementary Section S4. When data from the DXA-derived body composition indices were applied, it was found that the patients with a MELD-Na < 15, compared to those with a MELD-Na score ≥ 15, had significantly higher right leg lean mass (7873 vs. 5920 g, *p* = 0.04), percentages of fat in the (a) trunk (33% vs. 29%, *p* < 0.001), (b) right and left arms (38.4% vs. 35.5%, *p* = 0.008 and 39% vs. 35%, *p* = 0.007, respectively), and (c) right and left legs (37.7% vs. 35%, *p* = 0.02 and 37% vs. 34%, *p* = 0.007, respectively), as well as a higher amount of (a) left (1593 vs. 1357 g, *p* = 0.03) and right (1690 vs. 1438 g, *p* = 0.04) arm fat mass and (b) visceral (710 vs. 622 g, *p* = 0.035) and subcutaneous (1630 vs. 1370 g, *p* = 0.016) fat mass (Table 4). Finally, the patients with a MELD-Na ≥ 15 were frailer and displayed a lower physical performance (i.e., lower SPPB) and TSF, compared to those with a MELD-Na < 15 (Table 4).

In multivariable logistic regression analysis (excluding the components of MELD-Na score), a previous history of HE (OR 7.01, 95%CI: 2.74–18.1, *p* < 0.001), as well as lower albumin (OR 0.81, 95%CI: 0.73–0.87, *p* < 0.001), fibrinogen (OR 0.995, 95%CI: 0.991–0.998, *p* = 0.005) and trunk fat percentage (OR 0.92, 95%CI: 0.87–0.98, *p* = 0.007) were the only factors independently associated with a MELD-Na score ≥ 15.

### 3.3. Factors Associated with the Outcome: Univariate and Multivariate Analysis

During a median follow-up of 12 (range: 4–78) months, 96 (28.5%) patients died and 45 (13.4%) patients underwent LT, while 196 (58.2%) patients were still alive at the end of follow up. Univariate Cox regression competing analysis (death as the primary outcome and LT as the competing event) of clinical, laboratory and body composition parameters are presented in Table 5 and Supplementary Section S5.

Based on this analysis, the CTP score [Hazard Ratio (HR): 1.26, 95%CI [1.18–1.36], *p* < 0.001] and MELD-Na score (HR: 1.14, 95%CI [1.11–1.18], *p* < 0.001) were associated with mortality (Table 5). Regarding body composition, the lean mass of the right leg (HR: 0.98, 95%CI [0.96–0.99], *p* = 0.017), as well as percentages of trunk fat (HR: 0.96, 95%CI [0.93–0.98], *p* = 0.002) and left arm fat (HR: 0.98, 95%CI [0.96–0.99], *p* = 0.04) were predictive factors of the outcome. Finally, among anthropometric characteristics and function tests, SPPB (HR: 0.82, 95%CI [0.73–0.93], *p* = 0.002) and TSF (HR: 0.96, 95%CI [0.94–0.98], *p* = 0.004) were also significantly associated with mortality.

In multivariable Cox regression competing analysis, including CTP and MELD-Na scores but excluding their components (i.e., INR, bilirubin, creatinine, Na and albumin), trunk fat percentage (HR: 0.95, 95%CI [0.914–0.996], *p* = 0.03) and MELD-Na score (HR: 1.078, 95%CI [1.05–1.16], *p* = 0.02) were the only factors independently associated with mortality, while the results were similar after adjusting for the grade of ascites (HR: 0.96, 95%CI [0.911–0.99], *p* = 0.035; HR: 1.06, 95%CI [1.03–1.14], *p* = 0.022). These findings were confirmed in men, in whom trunk fat percentage (HR: 0.92, 95%CI [0.88–0.96], *p* = 0.01) and MELD-Na score (HR: 1.11, 95%CI [1.07–1.18], *p* = 0.03) were independent factors of the outcome, while MELD-Na (HR: 1.27, 95%CI [1.22–1.31], *p* = 0.02) score was the only factor independently associated with mortality in women. All anthropometric variables demonstrated VIF values below 5, indicating the absence of significant multicollinearity, while the sample size of our cohort, based on the number of events, was adequate for the primary analysis (Supplementary Section S6).

**Figure 1:**
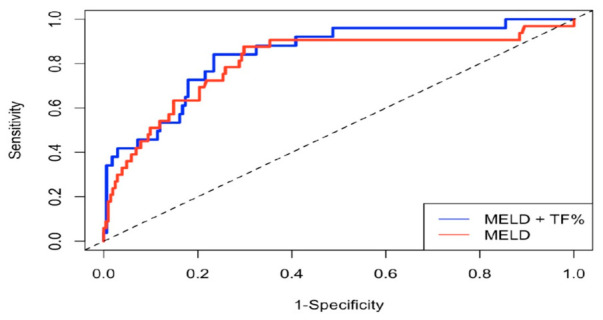
Time-dependent ROC curves for the discriminative ability of the model based on TF%/MELD-Na score and MELD-Sodium score to detect patient outcome in the whole cohort of patients with cirrhosis (*n* = 337 patients). MELD-Na: Model for end-stage liver disease-sodium, TF%: Trunk fat percentage.

**Figure 2:**
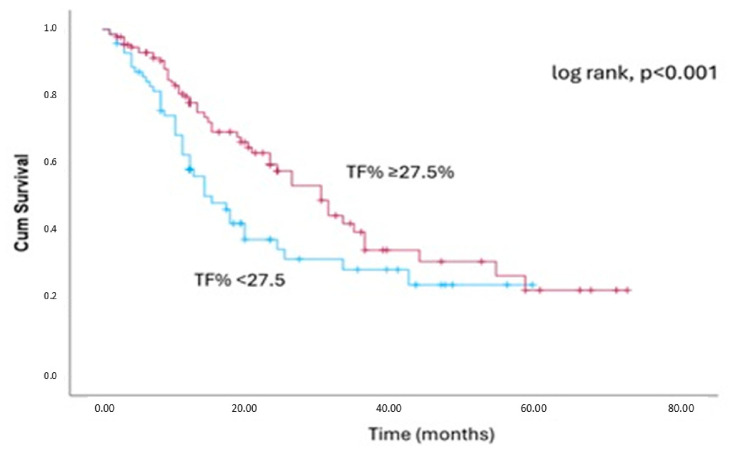
Kaplan–Meier curves with the best cut-off point of 27.5% of trunk fat percentage (log-rank, *p* < 0.001). MELD-Na: Model for end-stage liver disease-sodium, TF%: Trunk fat percentage.

In the total cohort, the model based on trunk fat percentage and MELD-Na score demonstrated good discriminative ability for predicting the outcome, with time-dependent AUC values of 0.84, 0.75, and 0.71 at 10, 20, and 30 months, respectively. Its performance was higher compared to that of the MELD-Na score (AUC: 0.80, 0.71 and 0.66, respectively), but the difference was not significant (*p* = 0.21) (Figure 1). The Harrell’s C-statistic was 0.80 and 0.77 for the new model and the MELD-Na, respectively, showing only a slight improvement. Finally, based on the best discriminative cut-off, the patients with a trunk fat percentage < 27.5% had a worse outcome (log-rank, *p* < 0.001) than those with a trunk fat percentage ≥ 27.5% (Figure 2).

## 4. Discussion

While prior studies have analyzed the association between different body composition measures and mortality in patients with cirrhosis, the main contribution of our study lies in the use of DXA-derived regional fat distribution, particularly trunk fat percentage, in relation to disease severity and outcomes. In fact, the impact of regional fat indices measured with DXA scans was evaluated regarding the outcome in a large cohort of patients with cirrhosis, taking into consideration other important prognostic factors such as total and regional lean indices, as well as performance status using well-established tools. The lower trunk fat percentage was (a) the only body composition index associated with the presence of decompensated cirrhosis, but this was not confirmed in multivariate analysis, and (b) independently associated with more advanced liver disease as measured by the MELD-Na score. In addition, the MELD-Na and trunk fat percentage were the only independent prognostic factors of the outcome, although separate analysis based on gender confirmed the prognostic impact of trunk fat percentage only in men, but not in women. In addition, the new score derived from these two parameters had good discriminative ability with numerically higher AUC values compared to the MELD-Na alone; however, this improvement was not statistically significant. Therefore, further studies in larger cohorts for external validation are needed for final conclusions before any incorporation of trunk fat percentage into prognostic scores or use in routine practice.

In daily clinical practice, the CTP and MELD-based scores are used for the evaluation of the prognosis of patients with cirrhosis, having satisfactory accuracy in predicting short-term survival. Nevertheless, these two prognostic scores are not without limitations, including the absence of consideration of important factors related to patients’ nutritional status, physical performance and frailty [7]. However, it is known that cirrhosis is a malnutrition state associated with insufficient dietary intake, abnormal intestinal absorption and metabolism dysregulation, leading to loss of skeletal and/or fat mass. In addition, other factors, such as chronic inflammation, inactivity, hyperammonemia, hormonal alterations and gut microbiota dysbiosis are implicated in alterations of body composition and the development of increased vulnerability and frailty [13].

Few literature studies, which focused mainly on the assessment of visceral and/or subcutaneous fat mass at the third or fourth lumbar vertebra with CT scanning, have evaluated the prognostic impact of adipose tissue in patients with cirrhosis [24], showing that reduced fat mass (adipopenia) in these regions is associated with adverse outcomes. Although adipopenia in other anatomical regions, such as arms and legs, has been associated with worse survival in several extra-hepatic clinical conditions [25], only recently has De Luca et al. [26] evaluated 110 patients with cirrhosis and found that low midarm fat was independently associated with a higher risk of spontaneous bacterial peritonitis development and death. However, it should be mentioned that this study [26] has several limitations considering the relatively small number of patients and the sub-optimal method used for the assessment of arm fat (TSF and midarm circumference). Importantly, no evaluation of other anatomical regions was performed, and the impact of functional performance or frailty was not included in the survival analysis [26].

Our study is the first one in which regional fat and muscle indices were evaluated in a large cohort of patients with cirrhosis using whole body DXA (which can accurately measure total and regional adiposity), while physical functionality was assessed based on measurements, such as SPPB and LFI. Generally, as expected, females, compared to males, had significantly higher fat but lower muscle indices (Table 2). These sex-related differences in body composition resemble those in the general population [27] and although cirrhosis is linked to reduced testosterone and increased estrogen levels, these hormonal shifts seem to occur proportionally across both sexes [28,29].

In addition, when the total cohort was divided based on MELD-Na score, in the univariate analysis all indices reflecting regional fat percentage (i.e., in the trunk and the four limbs) and the amount of fat mass in the left and right arms were inversely correlated with severity of liver disease (Table 4). In multivariate analysis, low trunk fat percentage reflecting relative trunk adipopenia, i.e., the reduced fat percentage located in the trunk as assessed by DXA, was the only body composition-related variable significantly associated with a MELD-Na score ≥ 15. Similarly, low trunk fat percentage was the only anthropometric index associated with the presence of decompensated cirrhosis (Table 3), although this finding was not confirmed in multivariate analysis. Moreover, we evaluated the prognostic impact of several clinical and laboratory variables, regional fat and lean indices, and anthropometric characteristics and tests relevant to physical performance and frailty. Interestingly, in multivariate Cox regression competing analysis, the percentage of trunk fat and MELD-Na score were the only factors independently associated with mortality, although separate analysis based on gender confirmed this finding only in men, but not in women. Based on these results, it could be suggested that the percentage of trunk fat (i.e., the percentage of the visceral and subcutaneous fat located between the chin and the hip joints) may have potential prognostic relevance in patients with cirrhosis. Although there is no clear explanation regarding this finding, it could be suggested that trunk fat percentage might be affected to a greater extent by the hypercatabolic state of cirrhosis compared to other body regions, or might be the result of endocrine dysregulations observed in cirrhosis (e.g., low testosterone levels may affect the appendicular more than trunk lean mass, while high adiponectin levels have been associated with the severity of cirrhosis and with a low trunk-to-appendicular fat ratio) [30,31,32]. Furthermore, although visceral and subcutaneous fat, as automatically assessed by DXA, were associated with the severity of cirrhosis, they had no prognostic impact, indicating that the entire trunk region might be more important regarding mortality. Interestingly, the SPPB and triceps skinfold were significant in univariate but not in multivariable analysis, where trunk fat percentage was the only anthropometric factor independently associated with the outcome. These findings indicate that trunk fat percentage may act as a surrogate marker of patients’ physiologic condition, as well as of energy reserves and chronic disease burden. The lack of the independent association of SPPB and triceps skinfold suggests that they may reflect similar aspects of body composition and frailty already represented by other measures.

Several literature studies have shown that reduced skeletal muscle mass is associated with higher complications rates and mortality before and after LT [7]. In all these studies, the evaluation of skeletal muscle mass was based on the assessment of CT cross-sectional areas at the level of the third lumbar vertebra, while few studies have evaluated the impact of muscle loss evaluated with DXA [7]. In addition, new prognostic scores combining skeletal muscle loss and MELD-Na score may improve the predictive ability of the MELD-Na score in LT candidates and after transjugular intrahepatic portal shunt placement [33,34]. In our cohort, the LMI and ALMI did not have a prognostic impact, and among muscle indices only the right leg lean mass was inversely associated with a MELD-Na score ≥ 15 and a worse outcome in the univariate analysis. However, these findings were not confirmed in multivariate analyses. These results could be attributed to the method used for muscle mass quantification (DXA scan), since CT scanning is considered a more objective method for muscle mass evaluation compared to DXA [35]. Thus, although an effort was made to eliminate the effect of fluid retention and ascites, the use of DXA to assess body composition, and/or other factors such as the prevalence of decompensated cirrhosis and gender distribution in our cohort, may have partially attenuated the prognostic impact of sarcopenia-related indexes. Nevertheless, our findings are in accordance with previous studies where CT scans were used for the evaluation of visceral and/or subcutaneous fat, in which adiposity, compared to muscle mass, had stronger prognostic impact [36,37,38], indicating the importance of the evaluation of fat indices in patients with cirrhosis. This suggests that fat distribution, and particularly trunk fat, could potentially have metabolic and inflammatory alterations that could not be fully reflected by muscle mass alone and probably partly explain the differences seen between adipopenia and sarcopenia-related measures in our study.

We acknowledge that our study has some limitations, since it is a single-center study including mostly patients with decompensated cirrhosis, thus impacting the generalizability of our findings. In addition, we measured muscle mass with DXA instead of CT scans. Nevertheless, DXA is a widely accepted method used in patients with cirrhosis and is usually preferred due to less radiation exposure for the patient, lower cost (compared to CT scans), and less interobserver variability compared to anthropometry methods, including triceps skinfold [18,19]. In addition, while dehydration or excess water retention may affect the accuracy of DXA measurements, the effect of hydration status is generally small regarding the evaluation of fat indices, and DXA is considered one of the most accurate methods for assessing total and regional body fat in several clinical settings [39,40,41]. Nevertheless, an effort was made in all patients for the optimal management of ascites before performing DXA. Moreover, given the observational nature of our study, we mainly showed associations between body composition and severity/prognosis of cirrhosis, and thus our findings might have been influenced by confounding factors which we could not evaluate, making it difficult to establish causality. Finally, it must be noted that the smaller number of women and the difference in body composition between men and women may have influenced the gender subgroup results and therefore a formal interaction analysis between sex and trunk fat percentages is needed to confirm a true effect. In conclusion, this is the first study in the literature evaluating the impact of regional muscle and fat indices, physical function, and frailty on the outcome of patients with cirrhosis. Our results suggest that the percentage of trunk fat is associated with the severity of liver disease and might be a promising prognostic biomarker of patient outcome, while separate analysis based on gender confirmed this finding only in men, but not in women. Nevertheless, further studies with external validation are needed to confirm our findings and to optimize the management and prognosis of patients with cirrhosis before and after LT.

## Figures and Tables

**Table 1:** Baseline characteristics of 337 patients with cirrhosis.

Variable	Patients, *n* = 337
Age, years, (SD)	56 (11)
Sex, males (%)	225 (67)
Etiology of cirrhosis, *n* (%)	
Viral hepatitis	86 (25)
Alcohol	96 (28)
MASLD/cryptogenic	37 (11)
PSC/PBC	64 (19)
Other causes	54 (17)
History of complications, *n* (%)	
Gastrointestinal bleeding	72 (21)
Hepatic encephalopathy	83 (24)
Spontaneous Bacterial Peritonitis	41 (12)
Ascites at baseline, *n* (%)	187 (55.5)
Hepatocellular carcinoma, *n* (%)	31 (9)
Child-Turcotte-Pugh score, (SD)	7.5 (2)
MELD-Na score, (SD)	15 (6.5)
BMI, kg/m^2^ (SD)	25 (5.5)
Trunk fat (%/mass, g), (SD)	31.8 (7)/13,100 (3210)
Estimated Visceral/Subcutaneous Adipose Tissue, g, (SD)	677 (290)/1522 (670)
Trunk lean mass, g, (SD)	26,373 (10,962)
Left leg fat (%/mass, g), (SD)	36 (8)/4587 (1490)
Left leg lean mass, g, (SD)	7112 (2081)
Left arm fat (%/mass, g), (SD)	38 (11)/1587 (450)
Left arm lean mass, g, (SD)	2105 (760)
Right leg fat (%/mass, g), (SD)	37 (8)/4670 (1520)
Right leg lean mass, g, (SD)	7075 (2019)
Right arm fat (%/mass, g), (SD)	37.8 (11)/1661 (510)
Right arm lean mass, g, (SD)	2273 (785)
Fat mass index (fat mass per height squared), kg/m^2^, (SD)	9.2 (4)
Lean mass index (lean mass per height squared), kg/m^2^, (SD)	16.3 (3.3)
ALMI (appendicular lean mass per height squared), kg/m^2^, (SD)	6.5 (1.4)
Short physical performance battery, (SD)	10.5 (2)
Liver frailty index, (SD)	3.9 (0.7)
Triceps skinfold, cm, (SD)	15.2 (6)
Midarm muscle circumference, cm, (SD)	22.5 (4.5)
Hand grip strength, kg, (SD)	20.5 (9)

ALMI: Appendicular lean mass index, MASLD: Metabolic dysfunction-associated steatotic liver disease, MELD-Na: Model for end-stage liver disease-sodium, PBC: Primary biliary cholangitis, PSC: Primary sclerosing cholangitis. SI conversion factors: To convert weight and mass from kg to g, multiply by 1000. To convert height, length, triceps skinfold, and midarm muscle circumference from cm to m, multiply by 0.01. To convert BMI, fat mass index, lean mass index, and ALMI from kg/m^2^ to g/m^2^, multiply by 1000. To convert fat mass, lean mass, trunk lean mass, appendicular lean mass, and limb lean/fat mass from g to kg, divide by 1000. To convert hand grip strength from kg to N, multiply by 9.80665. Continuous variables are expressed as mean (SD) for normally distributed data and median (range) for non-normally distributed data. Normality was assessed prior to analysis. Qualitative variables are expressed as counts with percentages.

**Table 2:** Characteristics of males and females in our cohort of 337 patients with cirrhosis.

	Males (*n* = 225, 67%)	Females (*n* = 112, 23%)	*p*-Value
Age, years	56.7 (11.1)	55.5 (10.8)	0.77
Etiology of cirrhosis, *n* (%)			
Viral hepatitis	56 (25)	30 (27)	0.87
Alcohol	64 (28)	32 (29)	0.79
Other causes	105 (47)	50 (44)	0.66
History of complications, *n* (%)			
Gastrointestinal bleeding	48 (21)	24 (22)	0.93
Hepatic encephalopathy	54 (24)	29 (26)	0.82
Spontaneous Bacterial Peritonitis	31 (14)	10 (9)	0.12
Hepatocellular carcinoma, *n* (%)	19 (8.5)	12 (11)	0.22
Creatinine, mg/dL, (SD)	1.01 (0.4)	0.74 (0.2)	<0.001
INR, (SD)	1.5 (0.5)	1.4 (0.5)	0.10
Bilirubin, mg/dL, (SD)	2.4 (0.24–40)	2.1 (0.3–32)	0.21
Albumin, g/dL, (SD)	3.4 (0.7)	3.5 (0.6)	0.16
Sodium, mmol/L, (SD)	136 (5)	137 (5)	0.17
Fibrinogen, mg/dL, (SD)	275 (88)	290 (91)	0.55
Ferritin, ng/mL, (SD)	210 (20–3377)	130 (5.7–1159)	0.15
Child-Turcotte-Pugh score, (SD)	7.8 (2.2)	7.2 (2)	0.21
MELD-Na score, (SD)	15.7 (6.5)	13.2 (5)	0.08
BMI, kg/m^2^ (SD)	26 (5.7)	24 (5.6)	0.033
Trunk fat (%/mass, g), (SD)	30 (7)/ 14,316 (1037)	34 (8)/ 11,189 (4200)	0.001/ 0.010
Estimated Visceral, (SD)/ Subcutaneous Adipose Tissue, g, (SD)	723 (272)/ 1635 (750)	587 (270)/ 1298 (540)	<0.001/ <0.001
Trunk lean mass, g, (SD)	29,289 (16,055)	20,652 (4852)	<0.001
Left leg fat (%/mass, g), (SD)	33 (7)/ 4333 (1723)	43 (6)/ 5022 (1730)	<0.001/0.08
Left leg lean mass, g, (SD)	7702 (1948)	5954 (1844)	<0.001
Left arm fat (%/mass, g), (SD)	33 (8)/1411 (599)	48 (11)/ 1723 (830)	0.004/ 0.11
Left arm lean mass, g, (SD)	2391 (664)	1545 (613)	<0.001
Right leg fat (%/mass, g), (SD)	34 (7)/ 4497 (1746)	43.8 (7)/ 5028 (1765)	<0.001/0.13
Right leg lean mass, g, (SD)	7678 (1898)	5892 (1711)	<0.001
Right arm fat (%/mass, g), (SD)	33 (11)/ 1572 (632)	48.6 (11)/ 1723 (760)	0.004/ 0.13
Right arm lean mass, g, (SD)	2577 (690)	1677 (601)	<0.001
Fat mass index (fat mass per height squared), kg/m^2^, (SD)	8.8 (3.9)	9.9 (3.4)	0.037
Lean mass index (lean mass per height squared), kg/m^2^, (SD)	17 (7)	14 (6)	<0.001
ALMI (appendicular lean mass per height squared), kg/m^2^, (SD)	7 (3.5)	5.8 (2.3)	<0.001
Short physical performance battery, (SD)	11 (2)	10.3 (2)	0.29
Liver frailty index, (SD)	3.8 (0.7)	4.01 (0.6)	0.12
Triceps skinfold, cm, (SD)	11 (5)	18.2 (7)	<0.001
Midarm muscle circumference, cm, (SD)	24.5 (7)	20 (6)	0.025
Hand grip strength, kg, (SD)	23 (9)	14 (6)	<0.001

ALMI: Appendicular lean mass index, MELD-Na: Model for end-stage liver disease-sodium. SI conversion factors: To convert weight and mass from kg to g, multiply by 1000. To convert height and length from cm to m, multiply by 0.01. To convert creatinine from mg/dL to μmol/L, multiply by 88.4. To convert bilirubin from mg/dL to μmol/L, multiply by 17.1. To convert albumin from g/dL to g/L, multiply by 10. To convert fibrinogen from mg/dL to g/L, multiply by 0.01. To convert ferritin from ng/mL to μg/L, multiply by 1. Continuous variables are expressed as mean (SD) for normally distributed data and median (range) for non-normally distributed data. Normality was assessed prior to analysis. Qualitative variables are expressed as counts with percentages.

**Table 3:** Characteristics of patients with compensated versus those with decompensated cirrhosis.

	Patients with Compensated Cirrhosis (*n* = 74, 22%)	Patients with Decompensated Cirrhosis (*n* = 263, 78%)	*p*-Value
Age, years	55.3 (12)	56.4 (10)	0.52
Sex, male, *n*, (%)	45 (61)	181 (68)	0.19
Etiology of cirrhosis, *n* (%)			
Viral hepatitis	21 (28)	65 (25)	0.44
Alcohol	24 (32)	73 (28)	0.38
Other causes	29 (40)	125 (47)	0.15
History of complications, *n* (%)	-		
Gastrointestinal bleeding	0 (0)	72 (27.4)	
Hepatic encephalopathy	0 (0)	83 (31.5)	
Spontaneous Bacterial Peritonitis	0 (0)	41 (15.6)	
Ascites at baseline, *n* (%) Grade: 1/2/3 *n* (%)	0 (0)	187 (71) 156(59)/31(12)/0(0)	-
Hepatocellular carcinoma, *n* (%)	6 (8)	25 (9.5)	0.71
Creatinine, mg/dL, (SD)	0.7 (0.2)	0.9 (0.5)	<0.001
INR, (SD)	1.2 (0.3)	1.5 (0.5)	<0.001
Bilirubin, mg/dL (range)	0.9 (0.24–1.5)	2.1 (0.29–40)	<0.001
Albumin, g/dL, (SD)	3.9 (0.7)	3.3 (0.5)	<0.001
Sodium, mmol/L, (SD)	139 (5)	136 (5)	<0.001
Fibrinogen, mg/dL, (SD)	330 (110)	280 (120)	<0.001
Ferritin, ng/mL	99 (5.7–510)	265 (15–3377)	<0.001
Child-Turcotte-Pugh score, (SD)	5.8(1.20	7.9 (2)	<0.001
MELD-Sodium score, (SD)	9 (3)	16 (6)	<0.001
BMI, kg/m^2^ (SD)	26 (5.7)	26 (5.6)	0.84
Trunk fat (%/mass, g), (SD)	33 (7)/12,591 (4986)	31 (7.5)/13,115 (5100)	0.012/0.30
Estimated Visceral/Subcutaneous Adipose Tissue, g, (SD)	691 (310)/1568 (750)	680 (276)/1508 (749)	0.84/0.61
Trunk lean mass, g, (SD)	27,499 (5230)	26,310 (5473)	0.41
Left leg fat (%/mass), g, (SD)	37 (8)/4600 (1452)	35 (80/4469 (1681)	0.07/0.58
Left leg lean mass, g, (SD)	7480 (1830)	6933 (1776)	0.54
Left arm fat (%/mass, g), (SD)	40 (12)/1734 (720)	38 (11)/1513 (610)	0.16/0.13
Left arm lean mass, g, (SD)	2390 (789)	1923 (679)	0.23
Right leg fat (%/mass, g), (SD)	38 (8)/4620 (1472)	36 (8)/4580 (1707)	0.11/0.37
Right leg lean mass, g, (SD)	7530 (1874)	6950 (1668)	0.29
Right arm fat (%/mass, g), (SD)	39 (12)/1760 (680)	38 (10)/1595 (625)	0.27/0.16
Right arm lean mass, g, (SD)	2365 (864)	2120 (706)	0.10
Fat mass index (fat mass per height squared), kg/m^2^, (SD)	9.4 (3.2)	9.1 (3.9)	0.61
Lean mass index (lean mass per height squared), kg/m^2^, (SD)	16.1 (2.4)	16.3 (3.4)	0.31
ALMI (appendicular lean mass per height squared), kg/m^2^, (SD)	6.5 (1.4)	6.6 (1.4)	0.95
Short physical performance battery, (SD)	10.7 (1.9)	10.1 (2.0)	0.04
Liver frailty index, (SD)	3.6 (0.7)	4.1 (0.6)	<0.001
Triceps skinfold, cm, (SD)	16.8 (10)	13.9 (7)	<0.001
Midarm muscle circumference, cm, (SD)	23 (5.6)	18 (8)	<0.001
Hand grip strength, kg, (SD)	22 (9)	19 (6)	0.16

ALMI: Appendicular lean mass index, MELD-Na: Model for end-stage liver disease-sodium. Continuous variables are expressed as mean (SD) for normally distributed data and median (range) for non-normally distributed data. Normality was assessed prior to analysis. Qualitative variables are expressed as counts with percentages.

**Table 4:** Characteristics of patients with cirrhosis and a MELD-Na score < 15 vs. those with a MELD-Na score ≥ 15.

	MELD-Na Score < 15 (*n* = 242, 71.4%)	MELD-Na Score ≥ 15 (*n* = 95, 28.6%)	*p*-Value
Age, years, (SD)	56.8 (11)	54.7 (11)	0.34
Sex, male, *n*, (%)	152 (63)	73 (77)	0.21
Etiology of cirrhosis, *n* (%)			
Viral hepatitis	56 (23)	30 (31)	0.17
Alcohol	66 (27)	30 (31)	0.45
Other causes	120 (50)	35 (38)	0.19
History of complications, *n* (%)			
Gastrointestinal bleeding	35 (14.5)	37 (39)	0.006
Hepatic encephalopathy	25 (11)	58 (61)	<0.001
Spontaneous Bacterial Peritonitis	21 (13.4)	20 (21)	<0.001
Hepatocellular carcinoma, *n* (%)	24 (10)	7 (7)	0.15
Creatinine, mg/dL, (SD)	0.8 (0.2)	1.2 (0.5)	<0.001
INR, (SD)	1.3 (0.2)	1.8 (0.6)	<0.001
Bilirubin, mg/dL, (range)	1.3 (0.24–23)	2.4 (0.29–40)	<0.001
Albumin, g/dL, (SD)	3.6 (0.6)	3.1 (0.5)	<0.001
Sodium, mmol/L, (SD)	138 (4)	134 (5)	<0.001
Fibrinogen, mg/dL, (SD)	318 (105)	244 (110)	<0.001
Ferritin, ng/mL	102 (5.7–3377)	288 (15–2558)	<0.001
BMI, kg/m^2^ (SD)	26 (5.4)	26 (4.8)	0.82
Trunk fat (%/mass, g), (SD)	33 (7)/13,216 (4894)	29 (6.5)/11,397 (4120)	<0.001/0.11
Estimated Visceral, (SD)/Subcutaneous Adipose Tissue, g, (SD)	710 (290)/1630 (780)	622 (240)/1370 (610)	0.035/0.016
Trunk lean mass, g, (SD)	25,700 (5936)	26,808 (11,500)	0.15
Left leg fat (%/mass), g, (SD)	37 (8)/4689 (1466)	34 (7)/4347 (1534)	0.007/0.29
Left leg lean mass, g, (SD)	7807 (1727)	6148 (1478)	0.16
Left arm fat (%/mass, g), (SD)	39 (11)/1593 (716)	35 (10)/1357 (673)	0.007/0.03
Left arm lean mass, g, (SD)	2426 (692)	1826 (490)	0.64
Right leg fat (%/mass, g), (SD)	37.7 (8)/4746 (1528)	35 (8)/4493 (1578)	0.023/0.15
Right leg lean mass, g, (SD)	7873(1726)	5920 (1448)	0.04
Right arm fat (%/mass, g), (SD)	38.4 (11)/1690 (700)	35.5 (10)/1438 (653)	0.008/0.04
Right arm lean mass, g, (SD)	2685 (787)	2003 (498)	0.45
Fat mass index (fat mass per height squared), kg/m^2^, (SD)	9.6 (4.1)	8.8 (2.9)	0.15
Lean mass index (lean mass per height squared), kg/m^2^, (SD)	16 (4.3)	16.7 (2.3)	0.99
ALMI (appendicular lean mass per height squared), kg/m^2^, (SD)	6.4 (1.8)	6.7 (1.1)	0.96
Short physical performance battery, (SD)	10.7 (1.5)	9.7 (2.3)	0.01
Liver frailty index, (SD)	3.8 (0.7)	4.2 (0.7)	0.003
Triceps skinfold, cm, (SD)	16.2 (7)	12.2 (5)	0.013
Midarm muscle circumference, cm, (SD)	23 (6.6)	21 (5.8)	0.16
Hand grip strength, kg, (SD)	21 (9)	18 (7)	0.07

ALMI: Appendicular lean mass index, MELD-Na: Model for end-stage liver disease-sodium. Continuous variables are expressed as mean (SD) for normally distributed data and median (range) for non-normally distributed data. Normality was assessed prior to analysis. Qualitative variables are expressed as counts with percentages.

**Table 5:** Univariate and multivariable analyses regarding the clinical and laboratory characteristics of 337 patients with cirrhosis associated with outcome (death as the primary outcome and liver transplantation as the competing event).

	Univariate Analysis			Multivariate Analysis		
	Hazard Ratio	95% Confidence Interval (Lower-Upper)	*p*-Value	Hazard Ratio	95% Confidence Interval (Lower-Upper)	*p*-Value
Age, years	1.2	0.99–1.51	0.15			
Sex, male, *n* (%)	0.9	0.95–1.77	0.71			
Cause of cirrhosis, *n* (%)	1.6	0.55–2.22	0.92			
Ascites	1.2	0.91–1.35	0.21			
Hepatocellular carcinoma	1.2	0.98–1.20	0.11			
Creatinine, mg/dL	1.89	1.31–2.71	<0.001			
INR	2.59	1.81–3.68	<0.001			
Bilirubin, mg/dL	1.13	1.08–1.18	<0.001			
Albumin, g/dL	0.92	0.91–0.95	<0.001			
Sodium, mmol/L	0.86	0.83–0.89	<0.001			
Fibrinogen, mg/dL	0.98	0.97–0.99	<0.001			
Ferritin, ng/mL	1.0	0.99–1.1	0.06			
Child-Turcotte-Pugh score	1.26	1.18–1.36	<0.001			
MELD-Na score	1.14	1.11–1.18	<0.001	1.078	1.05–1.16	0.02
BMI, kg/m^2^	0.99	0.96–1.026	0.71			
Trunk fat (%/mass, g)	0.96/1	0.93–0.98/1.0–1.0	0.002/0.92	Trunk fat %: 0.95	Trunk fat %: 0.914–0.996	Trunk fat %: 0.032
Estimated Visceral/Subcutaneous Adipose Tissue, g	1/0.99	0.98–1.11/0.98–1.01	0.29/0.15			
Trunk lean mass, g	0.99	0.98–1.11	0.23			
Left leg fat (%/mass, g)	0.98/1	0.96–1.005/1.0–1.0	0.12/0.69			
Left leg lean mass), g	1	1.0–1.0	0.085			
Left arm fat (%/mass, g)	0.98/1	0.96–0.99/1.0–1.0	0.04/0.63			
Left arm lean mass, g	1	1.0–1.0	0.45			
Right leg fat (%/mass, g)	0.98/1	0.96–1.005/1.0–1.0	0.13/0.94			
Right leg lean mass, g	0.98	0.96–0.99	0.017			
Right arm fat (%/mass, g)	0.98/1	0.96–1/1.0–1.0	0.06/0.12			
Right arm lean mass, g	1.0	1.0–1.0	0.66			
Fat mass index (fat mass per height squared), kg/m^2^	0.96	0.89–1.02	0.11			
Lean mass index (lean mass per height squared), kg/m^2^	0.99	0.97–1.02	0.37			
ALMI (appendicular lean mass per height squared), kg/m^2^	0.97	0.87–1.083	0.63			
Short physical performance battery	0.82	0.73–0.93	0.002			
Liver frailty index	1.45	0.92–2.29	0.12			
Triceps skinfold	0.96	0.94–0.98	0.004			
Midarm muscle circumference, cm	0.98	0.95–1.012	0.25			
Hand grip strength, kg	0.99	0.98–1.017	0.83			

ALMI: Appendicular lean mass index, MELD-Na: Model for end-stage liver disease-sodium.

## Data Availability

The data will be made available only upon reasonable request.

## Supplementary Materials

The following supporting information can be downloaded at: https://www.mdpi.com/article/10.3390/jcm15103697/s1.

## Abbreviations

The following abbreviations are used in this manuscript:

ALMI	Appendicular lean mass index
AUC	Area under the curve
BMI	Body mass index
CT	Computed tomography
CTP	Child-Turcotte-Pugh
DXA	Dual-energy X-ray absorptiometry
FMI	Fat mass index
HCC	Hepatocellular carcinoma
HE	Hepatic encephalopathy
LFI	Liver frailty index
LMI	Lean mass index
LT	Liver transplantation
MAMC	Midarm muscle circumference
MELD	Model for end-stage liver disease
MELD-Na	Model for end-stage liver disease-sodium
MRI	Magnetic resonance imaging
Na	Sodium
SD	Standard deviation
SPPB	Short Physical Performance Battery
TSF	Triceps skinfold
