# Supplementary material for: Relative Trunk Adipopenia Is Associated with the Severity of Liver Disease and Outcome in Patients with Cirrhosis
Source: J Clin Med. 2026 May 11;15(10):3697. doi: 10.3390/jcm15103697 (PMC13207654; doi:10.3390/jcm15103697)
Supplement: Supplementary file 1 [file jcm-15-03697-s001.zip › jcm-4254975-supplementary.pdf]

Relative Trunk Adipopenia Is Associated with the Severity of Liver Disease and Outcome in Patients with Cirrhosis

Aikaterini Kamiliou [1], Magdalini Adamantou [1], Eleni Pergantina [1], Nikolaos Rachiotis [1], Triada Bali [1], Dimitrios Mouziouras [2], Dimitra Lakiotaki [2], Vasileios Lekakis [2], Alexandra Alexopoulou [3], George V. Papatheodoridis [2] and Evangelos Cholongitas [1,2,*]

1 First Department of Internal Medicine, Medical School, National and Kapodistrian University of Athens, 11527 Athens, Greece; aikkamiliou@gmail.com (A.K.); magdalini_a@yahoo.gr (M.A.);
elpergantina@yahoo.gr (E.P.); nickrahiotis@gmail.com (N.R.); triadabali@gmail.com (T.B.)

2 First Department of Gastroenterology, General Hospital of Athens "Laiko", Medical School, National and Kapodistrian University of Athens, 11527 Athens, Greece; dimitris.200@hotmail.com (D.M.);
dlakiotaki@gmail.com (D.L.); lekakis.vas@gmail.com (V.L.); gepapath@med.uoa.gr (G.V.P.)

3 Second Department of Internal Medicine, "Hippokration" General Hospital of Athens, Medical School, National and Kapodistrian University of Athens, 11527 Athens, Greece; alexandra61@med.uoa.gr

* Correspondence: cholongitas@yahoo.gr; Tel.: +30-213-206-1643

Supplementary section S1: Patients' selection and inclusion/exclusion criteria

Participants were selected based on the presence of well-established cirrhosis, regardless of its etiology. The diagnosis of cirrhosis was based on liver biopsy and/or a combination of relevant clinical and radiological assessments. Decompensated cirrhosis was defined as a history of ascites, variceal bleeding, hepatic encephalopathy (HE) or non-obstructive jaundice in patients with cirrhosis. Exclusion criteria were a) age <18 years, b) acute liver failure, c) need for combined kidney and liver transplantation and d) previous LT. The presence of hepatocellular carcinoma (HCC) was not an exclusion criterion providing that there was underlying cirrhosis. Patients with decompensated cirrhosis were clinically stable regarding their cirrhosis, i.e. they had no cirrhosis-related complications [i.e. variceal bleeding, spontaneous bacterial peritonitis (SBP), HE] during the last month before baseline.

Supplementary section S2: Demographic, clinical and laboratory parameters collected at baseline of the study

For each patient included in the study the following information were collected: age, sex, etiology of cirrhosis, prior complications associated with cirrhosis (e.g. variceal bleeding, HE, SBP), comorbidities, and current medications. Blood samples were collected from all subjects including full blood count and standard biochemical analyses (ferritin included) as well as coagulation profile (fibrinogen included).

Supplementary section S3: Baseline characteristics of patients included in the study

The mean CTP and MELD-Na scores were 7.5 (2) and 15 (6.5), respectively. The etiology of cirrhosis was alcohol in 28% and viral hepatitis in 25% of the patients. Previous history of variceal bleeding, HE, SBP and HCC were recorded in 21%, 24%, 12% and 9% patients, respectively.

Supplementary section S4: Characteristics of patients based on MELD-Na score

Patients with MELD-Na score ≥15, compared to those with MELD-Na <15, suffered more frequently from a previous history of cirrhosis-related complications **(Table 4)**. In addition, the latter group had significantly higher baseline sodium [138 vs 134mmol/L, *P*<0.001], albumin [3.6 vs 3.1g/dL, *P*<0.001] and fibrinogen [318 vs 244mg/dL, *P*<0.001], but lower bilirubin [median: 1.3 vs 2.4mg/dL, *P*<0.001], INR (1.3 vs 1.8, *P*<0.001], creatinine [0.8 vs 1.2mg/dL, *P*<0.001] and ferritin [median: 102 vs 288ng/ml, *P*<0.001] **(Table 4)**.

Supplementary section S5: Clinical and laboratory factors associated with the outcome: univariate analysis.

Among the clinical and laboratory parameters, INR [Hazard Ratio (HR): 2.59, 95%CI [1.81-3.68], *P*<0.001), creatinine (HR: 1.89, 95%CI [1.31-2.71], *P*<0.001), total bilirubin (HR: 1.13, 95%CI [1.08-1.18], *P*<0.001), sodium (HR: 0.86, 95%CI [0.83-0.89], *P*<0.001), albumin (HR: 0.92, 95%CI [0.91-0.95], *P*<0.001) and fibrinogen (HR: 0.98, 95%CI [0.97-0.99], *P*<0.001), were significantly associated with the outcome **(Table 5)**.

Supplementary section S6: sample size justification

Based on the observed multivariable effect of trunk fat percentage on mortality (HR:0.95 per 1% increase; 95%CI:0.91–0.99), this corresponds approximately to an HR of 0.70 per 1 standard deviation increase in trunk fat percentage (~7%). Schoenfeld's formula indicates that approximately 62 deaths would be required to achieve 80% power and 83 deaths for 90% power (α=0.05, two-sided). In our cohort, 96 deaths occurred among 337 patients, indicating that the study was sufficiently powered to detect the observed association.
